# Barriers to adolescents’ access and utilisation of reproductive health services in a community in north-western Nigeria: A qualitative exploratory study in primary care

**DOI:** 10.4102/phcfm.v12i1.2307

**Published:** 2020-07-08

**Authors:** Awawu G. Nmadu, Suraya Mohamed, Nafisat O. Usman

**Affiliations:** 1 Faculty of Community Health Sciences, School of Public Health, University of the Western Cape, Cape Town, South Africa; 2 Department of Community Medicine, Faculty of Clinical Sciences, Kaduna State University, Kaduna, Nigeria

**Keywords:** sexual and reproductive health services, adolescents, access and utilisation, barriers, qualitative study, Nigeria

## Abstract

**Background:**

There is a dearth of qualitative studies exploring in-depth barriers that adolescents face in accessing and utilising reproductive health services (RHS) in Nigerian primary healthcare centres.

**Aim:**

This study explored the barriers hindering adolescents’ access to and utilisation of RHS in primary healthcare centres.

**Setting:**

This study was conducted in three primary healthcare centres in Kaduna North Local government area, Nigeria.

**Methods:**

This study used an exploratory descriptive qualitative design. Fourteen adolescents and three RHS providers were selected and interviewed. The data collection methods included individual in-depth interviews with adolescents and key informant interviews with service providers. Interviews were conducted between January 2017 and April 2017. Thematic content analysis was used to analyse the data.

**Results:**

This study identified three thematic barriers to adolescent’s utilisation of RHS. These included individual, social and health system barriers. Individual factors included the following: inadequate knowledge about RHS and poor attitudes of adolescents towards RHS; social factors such as parental influence, community and religious norms, financial constraints and stigma; and health system factors such as poor attitudes of service providers and inconvenient health facility opening hours hindered adolescents from utilising RHS. Most prominent was the strong influence of the social factors that affected adolescents to the extent that they felt constrained to freely utilise RHS out of a sense of commitment to religious values.

**Conclusion:**

The findings highlight the need for the development of programmes that would foster collective responsibility for supportive environments within communities and health facilities for positive adolescent RHS experiences.

## Introduction

The World Health Organization (WHO) defines adolescents as young people between the age of 10 and 19 years, and they constitute about a sixth of the world’s population.^1^ Adolescence has been described as a time when young people engage in increased risk-taking behaviour that exposes them to many health risks.^2,3^ Adolescent sexual and reproductive health (ASRH) is a global public health concern. This is because sexual activity of adolescents has been on the increase in many countries around the world.^4^ However, globally, adolescents access health services less frequently than expected^5^ because of the various challenges in accessing reproductive health services (RHS).^6^ In addition, adolescents are poorly informed about how to protect themselves from pregnancies and sexually transmitted infections (STIs).^6^ Regional differences exist with adolescents in developing countries experiencing greater challenges. Research has shown that in many countries in sub-Saharan Africa (SSA), young people face significant barriers to receiving ASRH services resulting in the underutilisation of these services.^7^

In Nigeria, a significant population of adolescents is sexually active (with nearly half [48.6%] of adolescents aged 15–19 years being sexually active) and involved in unprotected sexual activities with multiple partners, exposing them to negative reproductive health consequences.^8,9^ Women in the North West region of Nigeria (where Kaduna State the study area is located) marry at a much younger age (15.8 years) as compared to the national average of 19.1 years.^10^ Men marry later than women, with the median age at first marriage among men aged 30–59 years being 27.7 years. The percentage of female adolescents marrying before age 18 has declined from 48% to 43% between 2013 and 2018.^10^ Similarly, the percentage of female adolescents aged 15–19 years marrying before age 15 has declined from 12% to 8% over the same time period.^10^ Nigerian adolescents are also confronted with cultural and social contexts that likely affect their access to and use of RHS.^11^ Sexuality matters are regarded as taboo for adolescents because sex is traditionally regarded as sacred and for the married only.^12^ Although the government of Nigeria decided that education related to sexuality was to be integrated into the national school curriculum in 1999, the significant challenge that continues to threaten its implementation is the opposition from religious organisations and conservative political interest groups. These groups believe that sexuality and HIV education encourage young people to experiment with sexual activity.^13^ These cultural and religious contexts result in adolescents being inadequately informed about sexuality matters and underutilisation of RHS.

Adolescent-friendly RHS (where adolescents are treated with respect and their confidentiality maintained) have been largely lacking in the country, especially in rural areas.^14^ Reproductive health services are provided mainly by the Nigerian government through maternal and child health programmes. These services are usually not specifically targeted to the needs of adolescents and health workers providing ASRH services are not adequately skilled.^12^ Statistics in Nigeria have shown that among adolescents, RHS coverage rates are low, new HIV infection rates are high, contraception usage is low and pregnancy rates are high.^8,15^

There is a dearth of qualitative studies exploring barriers that affect adolescents’ utilisation of RHS in Nigeria. Prior studies have reported an increased understanding of the challenges that adolescents face while accessing or utilising of RHS using a qualitative exploratory approach.^16^ The aim of this study was, therefore, to qualitatively explore the barriers that influence adolescents’ access to and utilisation of RHS in primary healthcare centres.

## Methods

### Study design

An exploratory descriptive design was used for this study and was appropriate for understanding in greater depth the experiences, perceptions and feelings of adolescents regarding barriers influencing adolescent access to and utilisation of the RHS in their local context.^17^

### Setting

This study was conducted in three urban government primary healthcare centres in Kaduna metropolis North-western Nigeria between January 2017 and April 2017. Kaduna North local government area (LGA) has an area of 72 km² and is a densely populated urban area with an estimated population of about 500 000. There are 12 primary healthcare centres and 62 private clinics in the LGA. Non-governmental organisations (NGOs) and religious institutions also provide RHS in the LGA. Numerous patent medicine vendors and chemists exist in the LGA which adolescents patronise because of their proximity to the people. All the primary healthcare centres in Kaduna LGA provide RHS which include family planning, antenatal, postnatal care, child delivery services and health education. Curative services such as the treatment of STIs are also provided. The services provided are not targeted specifically at adolescents.

### Selection of participants

This study drew on two study populations: (1) male and female adolescents between the ages of 15 and 19 years residing in and attending the primary healthcare centres in Kaduna LGA; (2) service providers of RHS at the selected primary healthcare centres.

Three primary healthcare centres were purposively selected in the LGA based on their proximity to schools. Even though purposive sampling would have been preferred, convenience sampling was used to select the first 14 adolescents (5 males and 9 females) between the ages of 15–19 years who were attending the primary healthcare centres between January and April 2017 to seek any health services. When the researcher attempted to recruit adolescents coming specifically for RHS, attendance was poor and therefore she resorted to recruiting from adolescents attending the primary healthcare centres who had accessed RHS at some point in time. A total of 14 in-depth interviews were conducted because the researcher was satisfied that saturation point had been reached. Three nurses were purposively sampled (one from each primary healthcare centre) for key informant interviews (KII). These were nurses who had worked for at least 2 years providing RHS.

### Data collection

Data collection methods included individual semi-structured individual interviews with adolescents and KIIs with the RHS providers. All interviews were conducted by the principal researcher (a community health physician with expertise in qualitative research) after discussing the study protocols with eligible participants. Interview guides were used for interviews which consisted of open-ended questions covering adolescent’s knowledge of and perceptions about RHS, their experiences with the RHS offered and the barriers encountered (Box 1).

BOX 1 In-depth interview guide for adolescents.

What do you understand by reproductive health services? Probe: can you tell me some of the reproductive health services that you know about?

Do you think that it is necessary for adolescents like you to be provided with reproductive health services? Probe: what are the reasons for your thought?

Can you please tell me about the reproductive health services available in your community? Probe: what kind of service is provided for adolescents such as yourselves?

Can you tell me about where these reproductive health services are offered? Probe: where can adolescents obtain these reproductive health services in your community?

Can you tell me if you have you ever accessed and used these services from any of the places that you mentioned? If yes, would you share your experience with the services offered?

In your opinion, what encourages adolescents like you to make use of reproductive health services?

In your opinion, what discourages adolescents like you from making use of reproductive health services?

How would you like to see adolescent reproductive health services offered?

*Source:* Nmadu AG. Access and utilization of reproductive health services among adolescents in Kaduna North local government, Kaduna State North-West, Nigeria [Master’s thesis]. Cape Town: University of Western Cape; 2017

Healthcare providers were interviewed about their perceptions about the quality of RHS provided and the barriers adolescents faced in accessing RHS (Box 2).

BOX 2 Key informant interview guide for health providers.

What reproductive health services do you provide to adolescents? Probe on type of services offered, opening time and how often.

What is your opinion on the use of the services by adolescents? What is your attitude towards promotion of contraceptives and provision for adolescents?

What is your opinion on the services provided? Probe whether adequate and accessible for the adolescents?

What are your experiences in promoting and providing these services?

Why do you think adolescents attend your services?

Do adolescents have preference for certain services over others? If so, why do you think so? Are there any services that adolescents do not make use of or had made use of and discontinued? If so, why do you think so?

What are the reasons you think for why adolescents have not accessed public RHS?

How do you think the services can be improved?

*Source:* Nmadu AG. Access and utilization of reproductive health services among adolescents in Kaduna North local government, Kaduna State North-West, Nigeria [Master’s thesis]. Cape Town: University of Western Cape; 2017

RHS, reproductive health services.

The interviews were conducted in a private room at the clinic and each interview took between 20 and 60 minutes. All interviews were audiotaped with the participant’s permission and conducted in English as the participants were all conversant in English. The recorded interviews were transcribed verbatim. Trustworthiness was applied in this current study by paying attention to credibility, dependability and transferability.^19^ The transcripts were coded by two researchers and a final consensus was reached later. Being a community health physician who interacts and works with adolescents in the community, the principal researcher was aware that it did have the potential to influence the research process, analysis and results. The participants’ perceptions of the researcher including her professional role as a health worker could have influenced the adolescent’s interactions with her, and also the information revealed by the adolescents. To avoid this respondent bias, the researcher emphasised the importance of truthful and accurate reporting of their experiences. The principal researcher was aware that her values and beliefs as a healthcare worker could influence her perception of the information that was shared by participants. To avoid researcher bias, a reflexive diary was kept throughout the research process to document reflections about the interviews which was also utilised for data analysis.

### Data analysis

The transcribed data were analysed using thematic analysis which included (1) familiarisation with the data, (2) generating initial codes, (3) searching for themes, (4) reviewing themes and (5) defining and naming themes.^18^ The transcriptions along with the initial codes produced by the main researcher were shared with the members of the research team for validation.

### Ethical consideration

Ethical approval was obtained from the University of the Western Cape Biomedical Research Ethics Committee (Ethics number-130416-050) and the Kaduna State Ministry of Health Ethics Committee. Informed written consent was obtained from adolescents aged 18 years and above, service providers and parents or guardians of adolescents younger than 18 years. Assent was obtained from the adolescents younger than 18 years. The parents of these younger adolescents consented to the study because they were aware that their wards were already receiving HIV-treatment services.

## Results

### Characteristics of the participants

Individual interviews were conducted with nine females and five males aged 15–19 years. Of the 14 adolescents, two were married and were females, three were in secondary school, while 11 were at various levels in tertiary institutions. Based on religious affiliation, eight were Christians and six Muslims (Table 1).

**TABLE 1:** Characteristics of the adolescents.

Location	Age (years)	Sex	Marital status	Religion	Educational status
Health facility A					
	18	Female	Single	Christian	Tertiary institution
	19	Female	Single	Christian	Tertiary institution
	18	Female	Single	Muslim	Tertiary institution
	19	Female	Married	Muslim	Tertiary institution
Health facility B					
	18	Male	Single	Muslim	Tertiary institution
	18	Male	Single	Muslim	Tertiary institution
	18	Female	Single	Christian	Tertiary institution
	19	Male	Single	Muslim	Tertiary institution
	19	Male	Single	Christian	Tertiary institution
Health facility C					
	16	Female	Single	Christian	Secondary school
	18	Male	Single	Christian	Secondary school
	17	Female	Single	Muslim	Secondary school
	19	Female	Single	Christian	Tertiary institution
	19	Female	Married	Christian	Tertiary institution

*Source:* Nmadu AG. Access and utilization of reproductive health services among adolescents in Kaduna North local government, Kaduna State North-West, Nigeria [Master’s thesis]. Cape Town: University of Western Cape; 2017

The healthcare providers were nurses and all were women (Table 2).

**TABLE 2:** Characteristics of the key informants.

Location	Age	Sex	Marital status	Religion
Health facility A	46	Female	Married	Christian
Health facility B	30	Female	Married	Muslim
Health facility C	49	Female	Married	Christian

*Source:* Nmadu AG. Access and utilization of reproductive health services among adolescents in Kaduna North local government, Kaduna State North-West, Nigeria [Master’s thesis]. Cape Town: University of Western Cape; 2017

The emerging themes revealed three distinct levels of influencing factors: individual, community and health system-level factors (Table 3).

**TABLE 3:** Themes and subthemes.

Themes	Subthemes
Individual level	Limited knowledge about reproductive health services Poor attitudes of adolescents towards reproductive health services
Social factors	Parental influence Community and religious norms Financial constraints Stigma
Health system factors	Poor attitudes of service providers Inadequate resources Inconvenient health facility opening hours

*Source:* Nmadu AG. Access and utilization of reproductive health services among adolescents in Kaduna North local government, Kaduna State North-West, Nigeria [Master’s thesis]. Cape Town: University of Western Cape; 2017

### Individual-level factors

Apart from the two married adolescents, most of the adolescents had limited knowledge of the different types of RHS available at health facilities. The most commonly used method of contraception was condoms, followed by contraceptive pills. There was poor knowledge about other methods of contraception among this group of adolescents:

‘Mostly, we guys use condoms to prevent conception from taking place … that is what we are familiar with and comfortable with.’ (Male, 18 years, single, Muslim)

The adolescents across religious affiliations generally displayed negative attitudes towards premarital sex and also the use of contraceptives because that was meant to be the norm. The majority of them did not consider sexual relationships acceptable at their age and therefore felt that contraception should be for married people, which deterred them from accessing such services:

‘The one [*RHS*] on sexual intercourse is not good for adolescents like us … condoms are good for some people but not for me because we are not up to age for sexual activities. It’s not meant for us, it’s meant for adults … husbands and wives [*married people*].’ (Female, 16 years, single, Christian)

Some of the adolescents feared to discuss their sexual health problems with anyone, while others felt more comfortable discussing RH issues with their friends rather than consulting staff at the clinics. Experiences of embarrassment also featured prominently as reasons why adolescents did not access RHS:

‘I felt very shy and embarrassed during the process of taking the [*vaginal*] swab.’ (Female, 18 years, single, Muslim)

The findings also showed that when faced with RH problems, some adolescents delayed taking action and only did so when these problems persisted or became serious. This was to avoid stigmatisation from family and the community based on prevalent social and cultural norms.

### Community-level factors

Both the adolescents and nurses identified cultural and religious norms as the most significant barriers that affected adolescents’ access to and utilisation of RHS. Cultural taboos were reported to prevent adolescents from openly discussing sex and reproductive health issues:

‘Adolescents do not normally talk freely to each other about contraceptives, it is a very secretive matter, and it is not culturally acceptable.’ (Female, 18 years, single, Muslim)

The adolescents admitted that religious beliefs inhibited discussions about and the utilisation of contraceptives among adolescents:

‘Like cases like contraceptives, the religion [*Islam*] does not allow it [*the use of contraceptives*], … or as an adolescent, you bring up the issue of contraceptives, and they will say you are spoilt [*immoral*], where did you learn it from?.’ (Female, 18 years, single, Muslim)

These beliefs made the adolescents themselves feel guilty when engaging in sexual activity and also when accessing RHS.

‘Like one of the pastors said, sexual intercourse before marriage is like robbery because you are stealing. It’s only when it is legal i.e. when you are married that you’ll require such [*SRH*] services.’ (Male, 18 years, single, Christian)

Female participants also expressed a preference for a service provider of the same sex, which was mainly influenced by cultural norms and negatively influenced their health-seeking behaviour. They were usually distressed when they had to be attended to by service providers:

‘My parents told me that I should never allow a man to touch me, so if I know that it is a male doctor in the hospital then I will never go there, it is better I die in silence.’ (Female, 18 years, single, Christian)

‘Yes, it is a big issue because many husbands would not want their wives to be examined by a male health worker and I prefer also to be seen by a female health worker.’ (Female, 19 years, married, Muslim)

Access to RHS was also related to the socio-economic background of the family. Some of the adolescents mentioned transportation costs and the ability to pay for services as barriers to accessing RHS:

‘My dad is the one that gives me transport money whenever I want to go to the health facility. When my dad doesn’t have money, I don’t go.’ (Female, 16 years, single, Christian)

‘They [*the husbands*] complain about the financial burden, which they can do without … Some of them discourage the young woman [*from accessing care*] because they want to avoid the expenses of giving them money to buy drugs [*medication*].’ (Female, 19 years, married, Muslim)

Health workers also affirmed that culture and religion affect utilisation of RHS by adolescents, but they all unanimously agreed that these services are important for adolescents:

‘The very first thing that comes readily to me is culture. It is the kind of setting we have found ourselves. You grow up in a house where everybody is saying don’t do this-don’t do this [*have sex*], it is wrong to do this, so culture one. Secondly, is religion, we are very religious openly, but we pretend a lot, so that’s second. Then thirdly, there is this issue of stigma, I don’t want to be labelled as having had abortion. I have issues with infection …, but then, my parents or anybody I meet in the hospital will say ‘she is sexually active’ and it is unheard of that I am not marry but I am having sex, but we do [*it happens*].’ (Health worker, 46 years, Christian)

‘Also, you know [*for*] some their religion does not encourage adolescents using contraceptives. Again, others may think people are watching them to see what they are doing in the reproductive health clinic, and so would not want to be seen in a negative light for coming to access reproductive health services.’ (Health worker, 49 years, Christian)

‘All [*reproductive health*] services are good for them [*adolescents*]. You tell them the implication of what you are doing [*risk taking behaviour*], because in life, as children grow, they are exposed to drugs, sex and other things.’ (Health worker, 46 years, Christian)

‘To me, it is good to promote and provide it [*contraception services*] for adolescents and the reason I say this is that if you don’t do it at your back they will do.’ (Health worker, 30 years, Muslim)

One of the providers expressed her personal thoughts that though abstinence is best for adolescents, those who cannot abstain should be provided with contraceptives:

‘For those that cannot endure [*abstain from sex*], for such children if they are not advised about family planning, their lives are at risk. So, to be on the safe side we invite the parents and have private discussions with them and if they consent, we provide them [*the adolescents*] with family planning services.’ (Health worker, 49 years, Christian)

### Health system-level factors

Adolescents raised concerns about lack of privacy and confidentiality at the primary healthcare centres they are not comfortable where members of the community can see them:

‘Well … because of what people will say adolescent don’t usually go to health facilities before you know it tomorrow the whole town will get to know what you went to see the doctor for. In cases of contraceptives or family planning, you will also have to go to where nobody knows you or you have to access them on your own without prescription [*from a hospital*].’ (Female, 18 years, single, Christian)

‘I would like to see a society where adolescents can access reproductive health services without having to think about what she is going to go through, what she is going through, who is going to look at her, who is going to insult her because she knows that everything that is going on is going to be between her and her doctor and is going to be confidential.’ (Female, 18 years, single, Christian)

Healthcare workers also confirmed the perceptions of adolescents about confidentiality:

‘Before they were not coming freely, but since now it is something that is confidential, they just come straight to me and I give them the health talk and they collect what they want to collect, most of them collect condoms.’ (Health worker, 49 years, Christian)

The adolescents complained about the negative attitude and behaviour of health service providers. This included unfriendly and hostile behaviour and not being considerate:

‘Health workers have a negative attitude towards unmarried adolescents’ use of family planning, they frown at it.’ (Female, 19 years, single, Christian).

One health worker also affirmed that being harsh is a turn off for adolescents.

‘[*Y*]ou know a child will never come out to say what he is doing especially when you follow him in a harsh way, but when you follow him with petting, you will get him to tell you the truth.’ (Health worker, 30 years, Muslim)

Some adolescents and the service providers identified the inconvenient opening hours of the clinics as a major hindrance to adolescents accessing RHS. Adolescents complained that the opening hours of the health facilities coincided with the times that they were at school:

‘I don’t know if they [*health workers*] can talk to the patients to ask what timing is convenient for them and also make some of the clinic days evening hours like for those of us going to school. Some of us usually miss school whenever it is clinic day.’ (Female, 19 years, single, Christian).

Some adolescents also complained of instances where they had gone to access RHS, but did not receive the medications prescribed to them because the medications were not available:

‘My concern is the medicine. There was a time I came and I couldn’t get any medication. I was prescribed, and I went out to buy for myself.’ (Male, 18 years, single, Muslim)

The nurses also identified the lack of infrastructure, equipment and educational materials to keep adolescents engaged while attending the clinic:

‘The main problem we have is lack of space, but if we have enough space and adolescents can be directed to access services in one place separate from the adults and privacy can be ensured, it is going to be very pleasant and ok.’ (Health worker, 46 years, Christian)

‘Provisions need to be made for audio-visual educational materials, TVs, infrastructures, equipment, games …’ (Health worker, 49 years, Christian)

The nurses identified the lack of staff as another challenge to the provision of adequate services for adolescents:

‘There is the issue of inadequate staffing, which puts a lot of strain on the health worker and does not allow us to dedicate enough time when attending to adolescents.’ (Health worker, 30 years, Muslim)

## Discussion

This study showed that 14.2% of adolescents were married which is not far from the national value of 11.5% of adolescents who were currently married based on the Nigeria Demographic and Health Survey (NDHS) of 2018.^10^ In contrast, however, our study sample revealed that 66% of the adolescents were in tertiary institutions which was much higher than the national value of 2.2%. This could be explained by the fact that the current study was carried out in an urban setting, while the majority of the populace in Nigeria reside in rural areas where residents have lower levels of education.

This study identified barriers to adolescents’ access to RHS across individual, community and health system contexts. These interrelated factors identified in this study negatively influenced the adolescents’ access to and utilisation of RHS. At the individual level, limited knowledge of adolescents about the types of RHS available, adolescents’ negative attitudes towards the use of contraceptives and certain behaviours of the adolescents themselves created barriers to their use of RHS. Such behaviours included shyness, not being comfortable with a health provider of the opposite sex, fear of discussing their sexual health problems and delay in seeking care. Reports from previous studies have also identified some of these barriers. For example, a study conducted in the Lao People’s Democratic Republic reported adolescents’ limited knowledge about available RHS which constituted a barrier to their use of RHS.^20^ This highlights the need to provide adolescents with correct and comprehensive information on SRH and RHS which can afford them life-long protective benefits. Collaboration with relevant stakeholders in adolescent health to disseminate RH information using a variety of mass media and information, education and communication materials have been used to address lapses in RH knowledge among adolescents.^6^ Schools can be used as avenues where comprehensive sexuality education can be taught.^21^

A study conducted in Pakistan reported adolescents who expressed fear about discussing their sexual health problems with anyone.^22^ Studies in Burkina Faso, Ghana, Malawi and India also reported on how adolescents were not willing to seek SRH services until the problem became severe^23,24^ as was found in the present study. The attitude and behaviour of a dolescents in the current study towards sex and contraception seemed to be a reflection of the community-level barriers to RHS access including cultural and family and community norms. The norms regarding the unacceptability of adolescent sexual activity and RHS in this study seem to be founded in broader religious norms which negatively affected adolescents’ decision to use RHS. Findings of a study conducted among adolescents in Ghana similarly reported how their religious doctrines frowned upon contraception and/or abortion and as such deterred them from seeking RHS.^25^ This feature was seen across adolescents of both religions in this study with no differentiation and this, in turn, contributed not only to unmet needs for SRH information but perceptions that adolescent sex and contraceptive use are ‘bad girl’ behaviours. This reflects not only the sense of powerlessness but also the seeming commitment to religious values.

The literature has shown that in many African communities individual behaviour is strongly influenced by family norms.^23^ It has been found that the association of adolescents utilising RHS with adolescents engaging in sexual activity has played a role in contributing to the stigmatisation associated with ASRH services.^26,27^ The International Conference on Population and Development (ICPD) of 1994^28^ where Nigeria was represented emphasised that ASRH services should safeguard the SRH rights of adolescents in addition to respecting their cultural values and religious beliefs. Further research is required on effective strategies to generate acceptance of ASRH services among gatekeepers in the community, such as parents and religious leaders, so that SRH rights of adolescents can be respected.

The WHO has recommended for global standards of quality healthcare services for adolescents. These include provision of adolescents with information about their health and where and when they can obtain health services; ensuring that parents and community members support the provision of health services to, and their utilisation by adolescents; provision of in-facility services, referrals and outreaches that fulfil the needs of all adolescents; demonstration by providers of the technical competence required to provide effective health services to adolescents; facilities operating convenient operating hours, having a welcoming environment and the required equipment and supplies needed to ensure effective service provision to adolescents; facilities collecting, analysing and using data on service utilisation and quality of care for quality improvement; and finally involving adolescents in the planning, monitoring and evaluation of health services and in decisions regarding their own care.^29^ There is also a need for making adolescents aware of their sexual and reproductive rights so that they have the power to make healthier choices for themselves. The importance of adolescents recognising that they can play an active role in their SRH by giving voice to their own needs and rights cannot be overemphasised.

The findings of the current study highlight the importance of the creation of a conducive social environment that is supportive of ASRH services with interventions focusing not exclusively on adolescents but also family and community norms around ASRH. There is a need to build relationships between adolescents and their parents who support and reinforce positive health-related behaviours of adolescents. Interventions can be implemented that target parents of adolescents such as parental education on parent–child communication and ASRH issues. A descriptive review of the effectiveness of initiatives to improve adolescents’ utilisation of ASRH services in low-income countries concluded that programmes that promote access to and uptake of ASRH services are most effective when adolescent-friendly facility-based approaches are combined with community acceptance and demand-generation activities.^16^ Unfortunately, this is currently lacking in Kaduna. The need for interventions aimed broadly at community members and institutions outside the family like in neighbourhoods, schools, churches and mosques cannot be overemphasised. Such interventions should include conducting community sensitisation campaigns and community ASRH education programmes. The purpose is to shift mindsets to accommodate modern-day changes such as access to social media which adolescents have and which might positively influence their thinking and behaviour about SRH.

The negative attitudes and behaviour of health workers seemed to reflect the attitude of the community. This contributed to adolescents in this study avoiding RHS at health facilities for fear of being reprimanded and misjudged. Previous studies have similarly reported about the negative effects of health professionals’ attitudes in hindering on adolescents’ utilisation of RHS. For example, a study conducted in Kwazulu-Natal, South Africa and a review of evidence regarding the attitude of health workers towards adolescent SRHS in developing countries reported hostile and judgmental attitude of health workers as deterrents to adolescents’ use of the services.^30,31^ It has been recommended that health workers should be specifically trained to provide services related to the SRH needs of adolescents.^16,32^ Other health system barriers such as lack of privacy, inconvenient opening hours and inadequate resources for the provision of RHS identified in this study have similarly been reported in previous studies.^33,34^ These findings indicate that health systems need interventions that can cater to the timing needs of in-school adolescents and also tackle resource barriers for the provision of efficient ASRH services. Interventions targeted at improvement of capacities of primary healthcare centres for provision of comprehensive adolescent-friendly RHS cannot be overemphasised. Guidelines such as the WHO guidelines for making health services adolescent friendly which defines national quality standards for improving ASRH services could be used to improve these services.^35^

## Limitations of the study

Some limitations were encountered in this study; given the qualitative nature of the study, the findings could not be generalisable to the entire population of adolescents in Kaduna because of the small sample size as the participants may differ from other populations and those who chose not to participate in the study. For instance, the level of tertiary education of the general population in Nigeria is 8.8%, while that of adolescents aged 15–19 years is 2.2%.^10^ In this study, 66% of the population was undergoing tertiary education. However, the study provides relevant insights for this setting which could be applied to similar settings. The use of convenience sampling to select adolescents was another limitation, given its greater risk of bias as compared to purposive sampling. However, it was difficult to find adolescents accessing ASRH services specifically at the selected primary healthcare centres. In addition, younger adolescents (aged 15–17 years) were lacking in the sample. The research topic is sensitive and therefore it was anticipated that response bias might arise. To overcome this limitation, the participants were reassured of confidentiality and anonymity and the importance of giving honest answers to obtain truthful information that could be used to improve and provide better ASRH services. The adolescents irrespective of their religious background were conservative and it required much prompting to get them to open up. The principal researcher being in a different age bracket from the adolescents might have influenced the responses of the adolescents. Given that the principal researcher was female might have also influenced the responses of the male adolescents.

Based on the study findings the authors proposed recommendations summarized in Box 3.

BOX 3 Recommendations of the study.

Adolescents need to be equipped with accurate and comprehensive SRH information which can provide life-long protective benefits.

Interventions should be targeted at community members and institutions outside the family such as in neighbourhoods, schools, churches, mosques and workplaces. Such interventions can include conducting community sensitisation campaigns and community adolescent health education programmes. These interventions are required to develop positive social norms and community support for adolescents to practice safer behaviours and access SRH information and services.

It is necessary to improve the capacities of primary healthcare centres to provide comprehensive adolescent-friendly RHS. Training should be provided for health workers to equip them with the current knowledge and practices needed to deliver adolescent-friendly health services.

The government should, in addition, provide adequate resources for the provision of Change to ASRH services. This should cut across human and material requirements such as adequate manpower, drug and contraceptive supplies and educational materials.

Adolescents should be involved in the designing, planning and implementation of ASRH programmes in their communities.

*Source:* Nmadu AG. Access and utilization of reproductive health services among adolescents in Kaduna North local government, Kaduna State North-West, Nigeria [Master’s thesis]. Cape Town: University of Western Cape; 2017

ASRH, adolescent sexual and reproductive health.

## Conclusion

The adolescents’ experiences in Kaduna of accessing and utilising SRHS were influenced by factors located within the adolescents and also beyond what they could control. They encountered barriers to utilisation of RHS which emerged across the three specified levels of the individual, social and health system-level factors. Adopting interventions that could address these interrelated factors can go a long way to strengthen services tailored to the needs of adolescents as well as address their SRH needs and rights. Future research is necessary for programmes that target adolescents within the community to provide community-based reproductive health education and culturally sensitive services.
